# The Impact of Contaminating Poly (Methyl Methacrylate) (PMMA) Bone Cements on Their Compressive Strength

**DOI:** 10.3390/ma14102555

**Published:** 2021-05-14

**Authors:** Jakub Szabelski, Robert Karpiński, Przemysław Krakowski, Józef Jonak

**Affiliations:** 1 Section of Biomedical Engineering, Department of Computerization and Production Robotization, Faculty of Mechanical Engineering, Lublin University of Technology, Nadbystrzycka 36, 20-618 Lublin, Poland; 2 Department of Machine Design and Mechatronics, Faculty of Mechanical Engineering, Lublin University of Technology, Nadbystrzycka 36, 20-618 Lublin, Poland; j.jonak@pollub.pl; 3 Chair and Department of Traumatology and Emergency Medicine, Medical University of Lublin, Staszica 11, 20-081 Lublin, Poland; przemyslaw.krakowski@umlub.pl

**Keywords:** bone cement, compression strength, saline, contamination, degradation

## Abstract

This study presents an analysis of the impact of doping bone cement with saline. The two-ingredient cement, made right before the surgery, is subject to various kinds of organic contaminants and liquids used in the operating area, such as saline used to cleanse or cool it, during the process of mounting the prosthesis or bone-filling procedures. The processes of experimental destructive testing and statistical analysis have shown that, depending on the degree of saline doping, the static compressive strength parameters may greatly improve (with a low degree of contamination) or significantly worsen (when the contamination degree is higher). The limit value of the degree of salt admixture was estimated (2%), with which no statistically significant differences were observed in the cement strength in relation to the strength of non-contaminated cement.

## 1. Introduction

In the present day, the advance of science remains fast-paced, especially in disciplines associated with medicine and human life. The need to save human lives, relieve pain, reconstruct parts of the human body after injuries, and, finally, cure those suffering from various kinds of local or systemic ailments using traditional methods [1] are the flywheels of progress both in medicine itself and many other disciplines that are subsidiary to medicine in terms of their classification, but which share its goals, such as material engineering and, in particular, the development of biomaterials [2,3,4]. Some biomaterials used to aid patients are bone cements. Their basic applications involve the mounting of hip and knee joint prostheses [5] and, in the area of spine surgeries, for vertebroplasty and kyphoplasty procedures [6,7,8,9]. The gold standard for today’s implant mounting procedures is the use of PMMA (poly methyl methacrylate) cements. The material itself is not exactly new. Its creation took place at the beginning of the 20th century, when it was used as a lighter substitute for traditional glass. However, it was its high biocompatibility that inspired attempts to use it in ophthalmology, dentistry and, ultimately, orthopaedics.

The key properties of today’s lighter PMMA cements are associated with the mechanical parameters that impact the way in which they are used and the purposes they may serve—those properties being, among others, compression strength and hardness [10,11]. Cements used to mount prostheses must be distinguished by a durability strictly corresponding to that stated by the ISO 5833:2002 standard: 70 MPa. However, ultimately, the durability of the prosthesis/cement/bone connection placed in the harsh environment of the human body is decided by many factors. The cement (and, as a consequence, the bond) may be subject to degradation on the basis of endurance due to, i.a., the appearance of biofilm/bacterial colonies on the inner surface of the bone, improper prosthesis mounting, or improper preparation of the cement mass [11]. Seeing as other materials, such as blood or saline solutions, may be present in varying amounts in the operating areas that bone cements may be used in, and their properties are not necessarily neutral for the cement itself, resistance to contamination is another important trait of bone cements. Temperature and the cement polymerisation time associated with it constitute other important factors since, on the one hand, high temperatures that may, depending on the composition, be as high as 70 °C (see Table 1 [12]) may cause damage to the surrounding tissues, while, on the other, short polymerisation times may prevent the proper filling of the marrow cavity, resulting in the improper mounting of the prosthesis. During polymerisation, cements are often cooled using systemically neutral saline solutions, which may further contribute to their contamination.

Negligence in providing the optimal conditions for mounting the prosthesis as durably as possible may lead to the necessity of performing revision surgeries. This comes with inconveniences and risks for the patient, significant costs related to repeating the operation, and a greater risk of loosening the prosthesis even further.

Saline may act as a contaminant during surgeries and may be treated as such, but its use as a deliberate addition in order to decrease the stiffness of the final cement product has also been analysed. The supposed reason for this is excessive stiffness. During the process of vertebra augmentation, an acknowledged method of treating patients with pathological vertebra compression fractures, PMMA-based bone cement is also used; however, its compressive stiffness is too high for its intended purpose. Due to the increased risk of further fractures following vertebral augmentation by way of vertebroplasty or kyphoplasty, attempts to reduce this stiffness were undertaken, yielding success [13]. Considering the above, in this study, an attempt was made to analyse the behaviour of bone cement in the presence of contamination of the cement mass with a 0.9% saline solution. The aim of the study is to assess the impact of contamination of bone cement mass with saline solution on its compressive strength. Moreover, an attempt was made to estimate the range of the saline addition to ascertain an amount that does not negatively affect the material’s strength properties.

## 2. Materials and Methods

### 2.1. Materials

Subjected to the experiments were samples made of Depuy’s CMW3 cement. This is a material that is widely used to mount prostheses in surgical practice. This cement is produced in a two-ingredient form. Supplied separately are the liquid pre-polymer and powder-form polymer. The detailed compositions of CMW3 cement are shown in Table 2.

### 2.2. Sample Preparation

**Figure 1:**
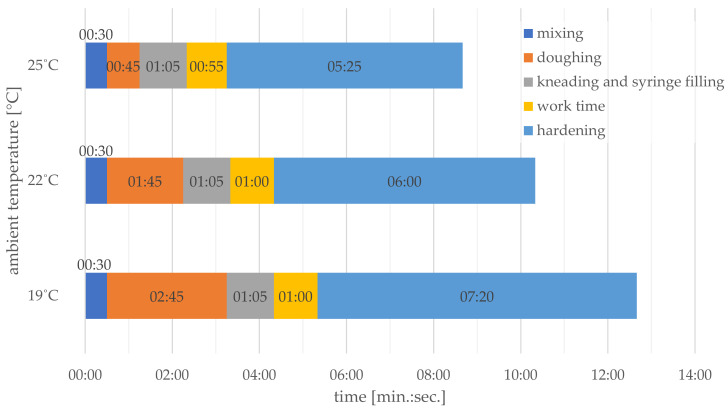
An example diagram of the impact of ambient temperature on the working time of the hand-mixed Palacos R cement placed in syringe.

The compression test specimens were prepared in accordance with international standard ISO 5833:2002 [14]. Joining and mixing the ingredients causes a highly exothermic polymerisation reaction to occur, in the course of which a hard, sturdy material is created in a relatively brief time (Figure 1 [12]).

During the hand mixing process, saline was introduced to the cement mass in the amount determined by the study plan, then spread in a regular manner within the body of the material being made. Cylindrical samples with dimensions of ⌀6 ± 0.1 mm × 12 ± 0.1 mm were made using a mould. In order to secure additional time for filling the moulds with the material before it hardened, both ingredients were pre-cooled to 16 °C. After the samples were produced, their dimensions were measured and, when the situation called for it, as per the standard, they were sanded using silicon carbide abrasive paper so as to make the top and bottom surfaces parallel, and the sample’s length compliant with the standard. The samples thus created were air–seasoned at a temperature of 23 ± 1 °C for at least 24 h; then, as per the ISO 5833:2002 standard, especially when it came to the compressive parameters, they were encumbered to the point of destruction.

### 2.3. Mechanical Testing

**Figure 2:**
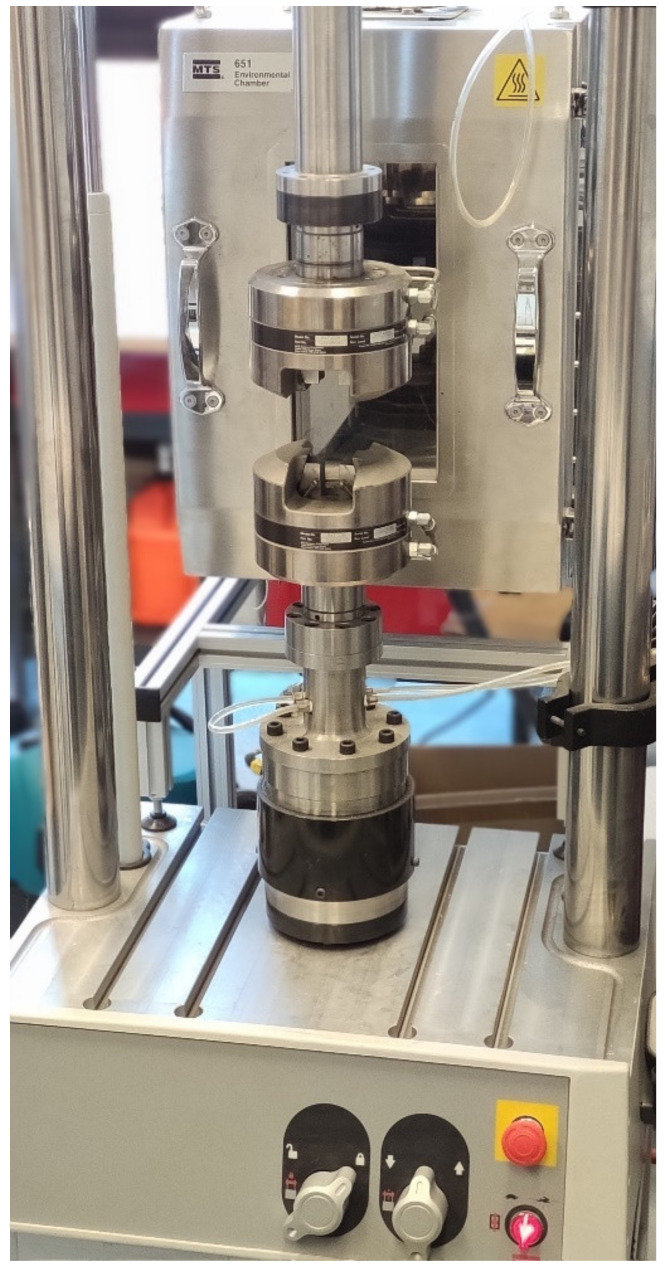
Test rig—MTS Bionix^®^ Servohydraulic Test Systems.

The compressive strength tests were performed with the use of the MTS Bionix–Servohydraulic Test System (Figure 2) (Eden Prairie, MN, USA) for biomedical material testing applications. The other component of the test set-up, the MTS TestWorks software (Eden Prairie, MN, USA), was employed to programme and execute the experiment procedure. The compression speed was specified as per the ISO 5388:2002 standard at 20 mm/min.

In order to mount the samples properly and ensure as pure a compression as possible, a dedicated mount was created, with its size adjusted to the cement samples’ dimensions.

The study was carried out in normal conditions, at 23 ± 1 °C. Notwithstanding that the standard required only 5 samples, more samples were considered in the experiment, so as to determine the values required with even more precision and to account for a potential margin, should any of the samples fall outside of the range given in the standard. During the study, the stress and displacement were recorded on a constant basis, along with the force values that resulted in the samples fracturing, deforming by 2% or reaching their yield point (whichever came earliest). The results were translated into internal stress parameters by dividing the force values obtained by the cross-sectional area of the cylindrical samples.

### 2.4. Statistical Analysis

A statistical analysis to determine whether there were statistically significant differences between the tested values of mean compressive strength was carried out using Statistica 13.3 (Tulsa, OK, USA). The program offers the user a set of the most important statistical methods, procedures and tools for data analysis, as well as a wide selection of the most advanced algorithms for modelling, forecasting, exploration and knowledge discovery in data sets, as well as excellent graphics and graphs that are not available in other programs [15].

Tukey’s HSD (honestly significant difference) test was used for the multiple comparison of several averaged groups and to separate homogeneous groups with statistically insignificant differences [16]. It is one of the few available post hoc tests, and the only one that allows for the testing of groups of unequal numbers, which was the nature of the results of the analysed strength. Due to the rejection of damaged samples, their number differs across the groups. Other multiple comparison test methods available in the Statistica software include Scheffé’s method, the Newman–Keuls method, Duncan’s test and Fisher’s LSD (least significant difference) test [17]. They also differ in the degree to which they obtain statistically significant results, and are separated into so-called liberal and conservative categories. “Conservative tests” are those where it is more difficult to obtain a statistically significant result and “liberal” tests are those where it is easier to obtain significant differences in averages [15].

The level of statistical significance in the study was α = 0.05.

## 3. Results

The average compressive strength values, depending on the amount of saline added, along with the determined standard deviation values, are presented in Table 3 and Figure 2. Clearly noticeable is the increase in average compressive strength at the lowest doping rate, equal to 1% by weight. Above this value, the average compressive strength would only drop. Of note are the small variation ranges (low standard deviation rates), which would suggest that the samples were made and examined properly, with the material’s behaviour remaining fairly consistent under different loads.

**Figure 3:**
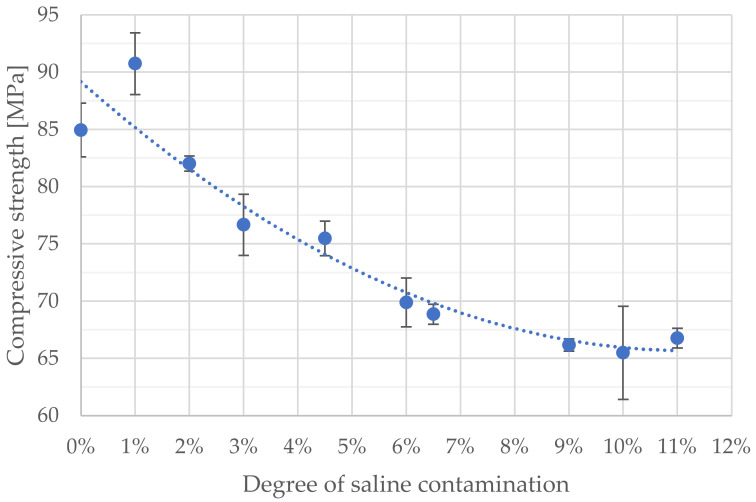
Impact of the degree of cement contamination on the average compressive strength.

Although the strength change progression graph (Figure 3) does show certain apparent changes, a statistical study was carried out using Tibco Statistica 13.3 software in order to check whether statistically relevant differences may be highlighted for the respective degrees of contamination. To this end, Tukey’s test was employed for the multiple comparison of an uneven number of samples in order to determine homogenous groups, i.e., groups that would feature average values that do not differ significantly between one another. The customary relevance level of α = 0.05 was adopted. As a result of the analyses, five groups were distinguished, as presented in Table 4. The important differences between the respective average strength values are presented as test results in Table 5.

## 4. Discussion

One way in which we can continue to achieve ever better PMMA-based bone cement properties is to dope the cements with substances that positively impact their desired performance parameters. The selection of materials subjected to testing is based on the knowledge of their properties and expected impact on the target material, as well as multiple static resistance and fatigue tests. Infectious complications following arthroplasty procedures for big joints are some of the most commonplace, and, at the same time, some of the most tragic complications following surgical procedures, not uncommonly requiring repeated revision surgeries and long-term antibiotic treatment [18]. Research on the matter has been ongoing for a long time, e.g., when it comes to the impact of adding antibiotics (gentamicin [19], flucloxalicin and vancomicin [20]) on the cement’s anti-bacterial properties in relation to the risk of infection as a complication following hip alloplasty [18] and attempts to reduce infection risk, while at the same time considering the potential negative impact of the medicines on the cement’s strength characteristics. The research has shown that, notwithstanding the addition of large amounts of antibiotics to the cement and their significant release into the surrounding tissues, doping leads to a significant decrease in the mechanical resistance of the encumbered cement. Interestingly, compressive strength decreases were not noted after a brief time following hardening, but were apparent and significant following 4 weeks of seasoning in BP. This was confirmed by, i.a., the increased cement porosity and lumping of the contrast agent after adding the antibiotics. The effects of adding an amphiphile phosphorylated 2-hydroxyethyl methacrylate (HEMA-P) molecule particle to commercial-grade cement were analysed as well. Its addition yielded positive results related to the proliferation and differentiation of osteoblast-like cells (SaOS-2), as well as very tight contact on the metal/cement boundary [21]. For the purposes of another work [22], the effects of adding 8% hydroxyapatite (HA) were examined, ultimately yielding an increase in the average tensile and compressive strength from, respectively, 20.40 to 25.20 MPa and from 84.04 to 89.57 MPa, with the hardening temperature dropping by approximately 3 degrees. Using a chain stopping agent, 1-dodecyl mercaptan (DDM) caused a steep hardening temperature decrease, with the drop amounting to approximately 30 degrees. Adding barium sulphate to cements as a contrasting agent for X-rays yielded an opposite/negative impact on the mechanical and thermic properties. The impact of adding silanated hyaluronic acid (as a coupling agent) on PMMA and polyethyl methacrylate (PEMA) cements was examined using Ringer’s solution in [23]. After 12 weeks of seasoning, a PMMA resistance of 16.6% (that of PEMA) was noted, with the biggest change noted in the PEMA cement with added silanated HA. The fatigue properties of PEMA cements decreased significantly after they were maintained in Ringer’s solution, with the biggest changes noted, once again, in the case of PEMA reinforced with silanated HA. This effect was attributed to the decrease in the silanated coupling agent’s efficacy in the presence of water. The PMMA cement’s fatigue resistance did not decrease following its immersion in the saline environment. The results of research entailing doping bone cements with Bisphosphonate, pamidronate (Pamifos 60), a medication used for treating, i.a., bone hypercalcaemia, bone tissue formation process disorders and osteolytic changes are known as well—a positive impact on bone formation has been observed, with bone growth increasing and an approximately 5% decrease in cement stiffness [24]. In the case of the results presented in this paper, the change in the compressive strength of bone cement with the addition of saline solution can be explained, as in [25], by the significant increase observed in the porosity of the obtained material.

As shown by the above study results, especially those of the statistical analysis performed, doping bone cements with saline has a significant impact on the change in their compressive strength. In the first phase, for the samples in the up to 1% saline addition by weight range, a statistically relevant compressive strength increase was recorded, which would point to the usefulness of such a procedure if one aims to obtain an even sturdier material. The limit value of the degree of salt admixture was estimated (2%), at which no statistically significant differences were observed in the cement strength in relation to the strength of non-contaminated cement. Further increases in the amount of saline in the material led to the ever-deepening weakening of its structure and properties. Contamination levels exceeding 6% resulted in compressive strength decreases below the hardened cement compressive strength value required, as per the ISO:5833 standard. It must be kept in mind that, in real-life conditions, the contamination of cement caused by the accidental release of outside materials into the cement’s structure will be of superficial character, contrary to the volumetric contamination of the cements analysed in the present study. It would seem that the obtained results related to the degree of degradation could be transposed to real-life conditions, though, as even superficial damage may lead to aseptic prosthesis loosening. This work assumes a maximum saline doping level of 11% according to the weight. Research upon greater levels of contamination appears to be pointless. However, it would be worth attempting to analyse the lower end of the range, especially in order to determine a saline doping level that would lead to achieving the maximum compressive strength.

The overall results obtained in this study may be of use in carrying out further analyses leading to the mathematical modelling of the function of strength variations in relation to the contamination level, such as in [26], or in attempts to utilise such models for predicting strength values with further contamination using machine learning/neural networks [26,27]. It must also be kept in mind that temporary compressive strength and its changes, along with the increase in contamination levels, may not be directly transposed onto real-life conditions, i.e., the additional impact of time and seasoning/the functioning environment of such materials. Thus, in the future, the above-described analyses should be expanded upon by those factors as well, as in [25,28].

What is important is that the modification examined above and the results obtained are analysed in the presented work only from the angle of static resistance; therefore, no global conclusions pertaining to the general improvement or worsening of the cement material may be drawn.

## 5. Conclusions

It has been shown that there exists a statistically relevant relation between the degree of doping bone cement with saline and its compressive strength. Its character is not even, i.e., an increase of 7% in the cement’s sturdiness was recorded with a small admixture of approximately 1% of weight. At a 2% admixture, no statistically significant differences were observed in the cement strength in relation to the strength of non-contaminated cement. Increasing the contamination level further caused the cement to weaken, with a level of approximately 6% yielding results below those required by the standard. Thus, the consideration of saline as a material to increase the sturdiness of the analysed material should be sought only in very small amounts of admixture according to the weight. Higher content levels should be treated as contamination, as they modify the studied properties of the cement in an undesirable manner. The likely cause of this is the introduction of empty spaces into the cement structure, i.e., pores that significantly degrade it. The results of the presented study should be treated as more of an introduction to further research aiming to determine the potential positive impact of saline on cement or, perhaps, the resistance of cement to excessive contamination from the angle of other performance parameters (such as cyclic load resistance). Thus, the focus should be placed on the range between 0 and 2 (or perhaps 3) percent, in order to determine the qualitative degree of sturdiness improvement more precisely.

## Figures and Tables

**Table 1:** The maximum temperature rise during cross-linking and cross-linking times of selected cements per ISO 5833.

Cement Type	Maximum Cross-Linking Temperature	Cross-Linking Time [min:s]
Boneloc	36 °C	11:00
Cemex RX	44 °C	13:20
Sulfix-6	48 °C	10:50
Zimmer LVC	52 °C	11:50
Palacos R Genta	56 °C	10:40
SamrtSet Genta	56 °C	09:50
Osteopal	58 °C	12:10
CMW Endurance	63 °C	12:10
CMW 3	65 °C	10:50
CMW 1 Genta	67 °C	09:10
Surgical Simplex Ro	69 °C	11:50

**Table 2:** Chemical composition of CMW 1 and CMW 3 bone cements.

Compound Name	CMW 3
**Bone Cement Powder Component**	
Polymethyl methacrylate	83.88
Benzoyl peroxide	2.00
Barium sulphate	10.00
**Bone Cement Liquid Component**	
Methyl methacrylate	97.5
N,N-dimethyl-p-toluidine	<2.50
Hydroquinone (ppm)	75

**Table 3:** Comparison of the average compressive strength values depending on the doping level.

Degree of Doping	Average Compressive Strength [MPa]	Standard Deviation
non-doped	84.95	2.35
1.0%	90.75	2.69
2.0%	82.02	0.66
3.0%	76.67	2.67
4.5%	75.48	1.50
6.0%	69.90	2.14
6.5%	68.86	0.88
9.0%	66.17	0.53
10.0%	65.50	4.07
11.0%	66.77	0.87

**Table 4:** Groups with statistically homogeneous compressive strength levels depending on the degree of cement contamination.

Contamination with Saline	Average Compressive Strength [MPa]	Homogeneous Groups				
		1	2	3	4	5
0%	84.95	x				
1%	90.75		x			
2%	82.02	x				
3%	76.67			x		
4.5%	75.48			x		
6%	69.90				x	
6.5%	68.86				x	
9%	66.17				x	x
10%	63.25					x

**Table 5:** Summary of the results of statistical analysis of the results of compressive strength tests depending on the degree of contamination.

Amount of Contamination	0% 84.95	1% 90.75	2% 82.02	3% 76.67	4.5% 75.48	6% 69.90	6.5% 68.86	9% 66.17	10% 63.25	11% 66.77
0%		0.00	0.27	0.00	0.00	0.00	0.00	0.00	0.00	0.00
1%			0.00	0.00	0.00	0.00	0.00	0.00	0.00	0.00
2%				0.00	0.00	0.00	0.00	0.00	0.00	0.00
3%					0.99	0.00	0.00	0.00	0.00	0.00
4.5%						0.00	0.00	0.00	0.00	0.00
6%							1.00	0.06	0.00	0.19
6.5%								0.38	0.01	0.72
9%									0.55	1.00
10%										0.29
11%										

## Data Availability

The data presented in this study are available on request from the corresponding authors.
